# Optical Properties and Thermal Stability of Ag-In-Cu Film on Aluminum Alloy Substrate Deposited by Magnetron Sputtering

**DOI:** 10.3390/ma18061318

**Published:** 2025-03-17

**Authors:** Xiaojun Zhao, Xinyue Wang, Ke Liu, Yuxiang Jiang, Zhenwu Peng, Yuchi Zhou, Zhonglin Qian, Wei Li, Lekang Lu, Lairong Xiao, Zhenyang Cai

**Affiliations:** 1 School of Materials Science and Engineering, Central South University, Changsha 410083, China; zhaoxj@csu.edu.cn (X.Z.); wangxyz127@163.com (X.W.); 31220148@csu.edu.cn (K.L.); jiangyuxiang1999@163.com (Y.J.); pzw799493@163.com (Z.P.); 13574117758@163.com (Y.Z.); 8204220210@csu.edu.cn (Z.Q.); 916lulekang@sina.com (L.L.); 2 State Key Laboratory of Powder Metallurgy, Ministry of Education, Central South University, Changsha 410083, China; 3 Powder Metallurgy Research Institute, Central South University, Changsha 410083, China; csuliw@csu.edu.cn; 4 State Key Laboratory for Light Weight and High Strength Structural Materials, Central South University, Changsha 410083, China

**Keywords:** reflectance, silver alloy films, thermal stability, magnetron sputtering

## Abstract

High-reflectivity metallic films on aluminum substrates are crucial in advanced aerospace and military applications due to their excellent reflectivity and workability. In order to further improve the reflectivity and thermal stability of films, this study investigated the deposition of AgInCu_x_ (x = 1, 3, and 5 wt.%) films on Al 6061 alloy substrates using magnetron sputtering, exploring the impact of deposition parameters and composition on their optical properties and thermal stability. Increased copper content improved thermal stability, while it compromised reflectivity. Additionally, increasing deposition power and time initially enhanced reflectivity, but beyond an optimal point, it decreased. Therefore, the AgInCu films deposited at 30 W for 2 min exhibited the highest reflectivity of 99.8% in the near-infrared range, making them promising candidates for reflective films in next-generation optical applications.

## 1. Introduction

Due to their low-cost, lightweight properties and excellent workability for complex curved surfaces, aluminum alloys are a new generation of reflective mirrors used in aerospace and military applications, such as space telescopes, remote sensing imaging, infrared detection unmanned aerial vehicles (UAVs), and solar power reflectors [1,2,3,4,5]. The continuous advancement of optical systems necessitates reflective mirrors with performance exceeding aluminum alloy with 92% reflectivity [6,7,8]. Therefore, high-reflectivity metallic films based on the aluminum substrate are widely employed due to their superior characteristics, including high reflectance, broad working wavelength range, and good electrical conductivity [9,10,11,12].

Silver is highly favored as a reflective material due to its high reflectance, low emissivity, and low polarization splitting [13,14,15,16,17]. However, silver is prone to agglomeration and reacts with halogen elements in the air, which degrades its optical performance [18,19,20]. To address these issues, researchers have explored doping silver with other metals to inhibit agglomeration, enhance thermal stability, and improve environmental resistance [6,21,22,23]. Cheng et al. [24] designed Ag-La films with an average reflectivity of 95%, which decreased to 81% after annealing at 400 °C. Compared with pure Ag films, the thermal stability of alloy films was improved while retaining high reflectance. Chen et al. [25] deposited AgIn_x_ (x = 0.5, 1, and 2 wt.%) films on silica glass substrates at room temperature using magnetron sputtering. The films with 0.5 wt.% In exhibited the highest reflectance of 99%. However, the reflectance of films decreased with increasing In content. Additionally, the reflectance after damp heat experiments was improved evidently as increasing In content. Wang et al. [26] prepared a structure of 10 nm ITO/100 nm AgIn/10 nm ITO films at different sputtering temperatures, finding that those deposited at room temperature achieved a maximum reflectance of 95.3%. In summary, indium can inhibit the migration of Ag atoms under a damp heat environment to improve thermal stability [26]. Furthermore, Yang et al. [27] synthesized Ag-Cu nanoparticles and verified that copper contributes to improved thermal stability due to its high melting point and low diffusion coefficient. However, there are few studies investigating the mechanism by which Cu content influences the thermal stability of the optical performances of silver alloy films.

Therefore, the AgInCu_x_ (x = 1, 3, and 5 wt.%) films were deposited onto the Al 6061 substrate via magnetron sputtering at room temperature. The effect of preparation parameters and Cu content on its optical performance and thermal stability was investigated. The research in this paper focused on fabricating high-reflective mirrors with thermal stability. The use of AgInCu_x_ alloys for reflective films has significant potential in optical applications.

## 2. Materials and Methods

### 2.1. Materials and Preparation

The sputtering targets were AgInCu_x_ (x = 1, 3, and 5 wt.%) disks with diameters of 50 mm, and the chemical composition are listed in Table 1. The aluminum 6061 alloys were cut into bulks with a dimension of 20 mm × 20 mm × 4 mm that were used as substrates. The aluminum substrates were processed using the ultra-precision single-point diamond machining technology (SPDT) to achieve a surface roughness of 6 nm. Then substrates were ultrasonically cleaned in anhydrous ethanol and acetone for 15 min, respectively, and dried in an oven.

AgInCu_x_ (x = 1, 3, and 5 wt.%) films were deposited onto the Al 6061 alloy by the JZCK-400DJ Magnetron Sputtering (MS) (Liaoning Juzhi Company, Shenyang, China) at room temperature. During the deposition, vacuum chamber pressure was maintained below 3 × 10^−3^ Pa, with a sputtering pressure of 0.7 Pa. Pure Ar gas (99.99%) was introduced into the chamber at a flow rate of 15 sccm. To investigate the influence of deposition parameters on the optical performance and thermal stability of films, the AgInCu_x_ (x = 1, 3, and 5 wt.%) films were deposited at varying deposition power and deposition time, as detailed in Table 2. After deposition, the films were exposed at 200 °C for 1 h under vacuum environment with 10^−1^ Pa pressure to evaluate the changes in film reflectance.

### 2.2. Characterization of Films

The phase composition of the films was analyzed by the Smartlab SE Grazing Incidence X-ray Diffraction (GI-XRD) (Rigaku Corporation, Tokyo, Japan) with Cu Kα radiation. The diffraction angle 2θ was varied from 20° to 90°, and the incident angle was set as 0.5° to mitigate substrate interference. The surface morphology of the layers was characterized by the AMBER GMH scanning electron microscopy (SEM) (TESCAN, Brno, Czech Republic). The surface roughness of the films was characterized by the Wyko NT9100 3D optical profilometer (Veeco Metrology Inc., Tucson, AZ, USA).

### 2.3. Optical Performance of Films

The thickness, refractive index, and extinction coefficient of the AgInCu_x_ (x = 1, 3, and 5 wt.%) film were measured with the HORIBA France SAS spectroscopic ellipsometry (HORIBA Scientific, Palaiseau, France). The reflectance in the range of 800 to 2500 nm was conducted with a Cary-5000 UV-Vis and Infrared Spectrophotometer (Agilent Technologies, Santa Clara, CA, USA) equipped with an integrating sphere.

## 3. Results

### 3.1. Phase Composition

**Figure 1:**
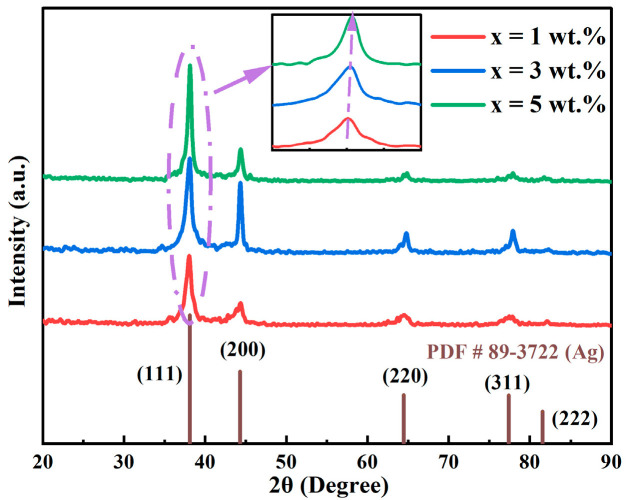
The XRD patterns of AgInCu_x_ (x = 1, 3, and 5 wt.%) films.

Figure 1 presents the XRD patterns of AgInCu_x_ (x = 1, 3, and 5 wt.%) films. The diffraction peaks were observed at 38.34°, 44.89°, 64.19°, and 78.35°, which correspond to the (111), (200), (220), and (311) crystal planes of Ag, respectively. The films exhibited a polycrystalline structure with a face-centered cubic (FCC) lattice, and the (111) crystal plane was the preferred orientation. Notably, the absence of significant In and Cu diffraction peaks was likely due to their low concentration and high solubility within the Ag matrix [25]. As the Cu content increased, the intensity of the (111) diffraction peak increased and the half-peak width decreased, indicating enhanced crystallinity of the films. This suggested that the formation of a solid solution with Ag, In, and Cu reduced defects and stress concentration. Furthermore, the diffraction peaks of (111) slightly shifted to the right with increasing Cu content. This was attributed to the reduction in lattice constant, resulting from the substitution of Ag atoms (atomic radius: 144 pm) with small Cu atoms (atomic radius: 128 pm) [28]. As the Cu content increased, the extent of this substitution also increased, which in turn caused a decrease in the lattice constant. In contrast, the constant In concentration had no significant impact on the lattice constant.

### 3.2. Surface Morphology

**Figure 2:**
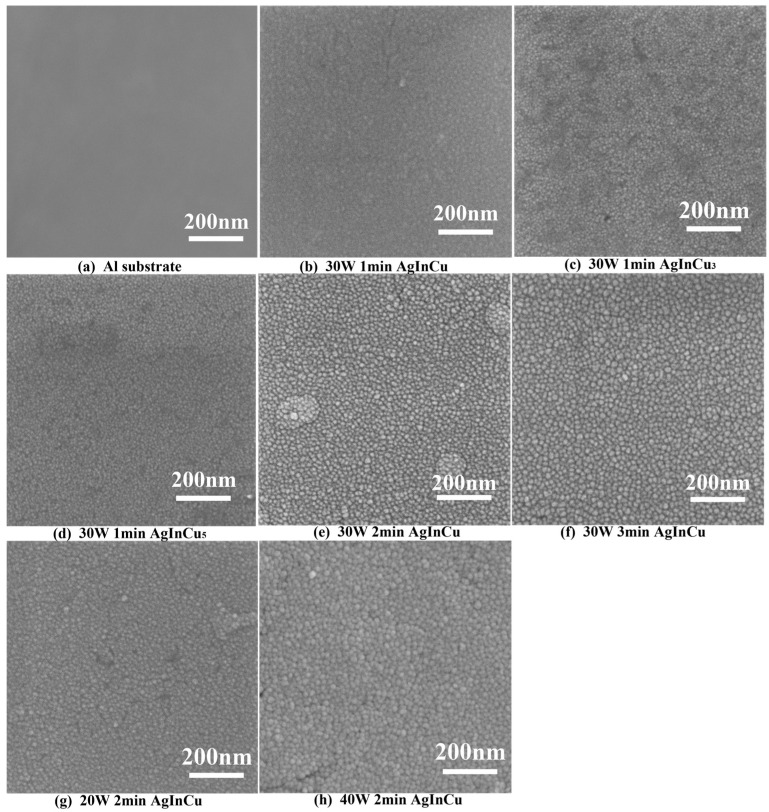
The SEM images of (**a**) Al substrate and (**b**–**h**) AgInCu_x_ (x = 1, 3, and 5 wt.%) film prepared at different parameters.

**Figure 3:**
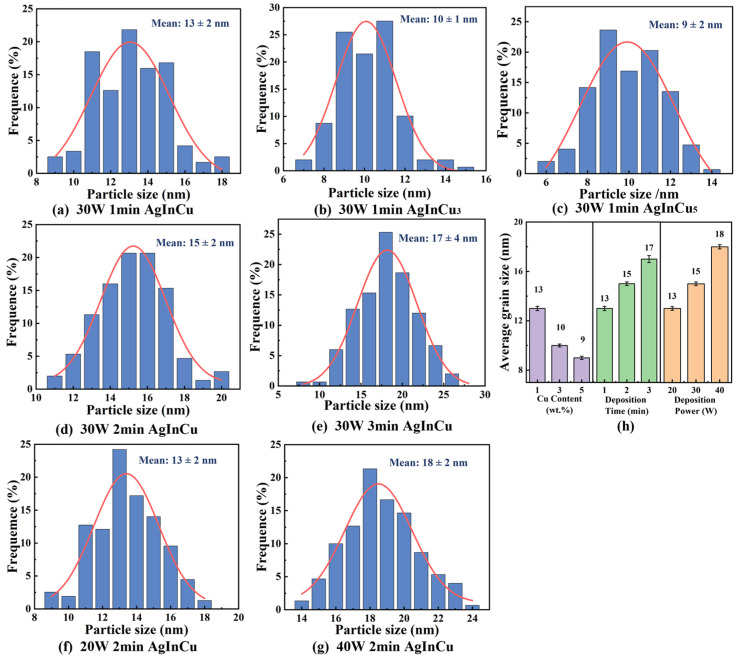
The (**a**–**g**) frequency distribution histogram of grain size in AgInCu_x_ (x = 1, 3, and 5 wt.%) film prepared at different parameters. (**h**) The average grain size of AgInCu_x_ (x = 1, 3, and 5 wt.%) films.

Figure 2a illustrates the surface morphology of the Al substrate after ultra-precision single-point diamond machining, revealing a flat surface with few defects. Figure 2b–h shows the microstructure of AgInCu_x_ (x = 1, 3, and 5 wt.%) films prepared at different parameters. In general, films exhibited a granular texture with different sizes and a flat, homogeneous appearance. Figure 3a–c presents the grain size of AgInCu_x_ films; the average grain sizes of the films were 13 nm, 10 nm, and 9 nm for x = 1, 3, and 5 wt.%, respectively. The grain size of the AgInCu_x_ films tended to decrease with increasing Cu content. One-way ANOVA confirmed a significant effect of Cu content on grain size (F(2, 413) = 152, *p* < 0.001). It seemed that Cu enhanced the nucleation sites to increase the number of crystal nuclei. Additionally, Cu hindered atom diffusion to constrain the growth of grain [29]. Therefore, the addition of Cu appeared to influence the grain size in the Ag alloy films, leading to a reduction in grain size.

According to Figure 3a,d,e, the AgInCu films deposited for 1 min, 2 min, and 3 min exhibited spherical grains with average diameters of 13 nm, 15 nm, and 17 nm, respectively. As the sputtering time increased, the films grew thicker from 32 nm to 104 nm, and the grain size increased. The ANOVA results showed a significant effect of sputtering time on grain size (F (2, 416) = 104.4, *p* < 0.001), indicating statistically significant differences. The increase in grain size with longer sputtering durations was primarily attributed to the augmented flux of incident atoms impinging upon the substrate. The thick film inherently provided great three-dimensional space for grain expansion [30]. Otherwise, the long sputtering time increased the number of incident atoms, enhancing the availability of adatoms and facilitating grain growth through absorbing atoms and small nuclei [31,32]. Therefore, the grain size showed a growing trend with prolonged sputtering time.

The AgInCu films deposited at 20 W, 30 W, and 40 W, as shown in Figure 3f,d,g, had average grain sizes of 13 nm, 15 nm, and 18 nm, respectively. Additionally, the ANOVA for sputtering time also showed a significant effect on grain size (F (2, 454) = 290.9, *p* < 0.001). It’s demonstrated that an increase in deposition power resulted in a corresponding increase in grain size. The formation and growth of films encompassed the absorption and diffusion of atoms, nucleation, and grain growth [31,32]. During the magnetron sputtering deposition process, positively charged argon ions bombarded the target material. The bombardment transferred sufficient kinetic energy to the target atoms, enabling their ejection from the target and deposition onto the substrate. These adatoms were absorbed onto the substrate and diffused along various directions. Subsequently, interatomic interactions facilitated the formation of atomic clusters. And the stable nuclei formed when these clusters reached a critical size and the total free energy decreased. These stable nuclei then continue to grow by absorbing adatoms and unstable nuclei, ultimately leading to the formation of stable grains [33,34,35]. The increasing sputtering power enhanced the energy of argon ions incident on the target atoms. Consequently, the atoms bombarding the substrate gained sufficient kinetic energy to overcome the diffusion activation energy, promoting grain growth and leading to larger grain sizes. Therefore, high sputtering power resulted in large grains.

### 3.3. Surface Roughness

**Figure 4:**
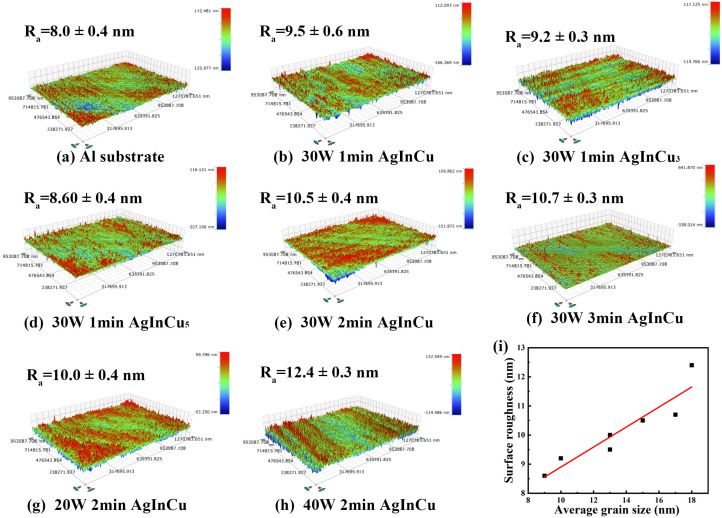
The surface roughness of (**a**) Al substrate and (**b**–**h**) AgInCu_x_ films prepared at different parameters and (**i**) surface roughness Ra (nm) vs. average grain size (nm) for AgInCu_x_ films.

The arithmetic average roughness (Ra) of Al substrates and AgInCu_x_ films deposited under various parameters are depicted in Figure 4. According to Figure 4a–h, the Al substrate exhibited a surface roughness of 8.0 nm, and the deposited films displayed an approximate roughness of 10 nm. The surface roughness of thin films significantly impacts their reflective properties. Due to the peaks and valleys, the large surface roughness could cause light scattering, reducing the light intensity in the specular reflection direction. Therefore, small surface roughness of thin films is crucial for minimizing diffuse reflection and scattering and effectively improving their reflectivity [17,36]. Analysis of the 3D surface topography highlighted that as the Cu content increased, the surface roughness decreased, as seen in Figure 4i. Furthermore, the surface roughness increased with increasing sputtering power and deposition time. These observations aligned with the relationship between grain size and deposition parameters. To further validate the relationship between surface roughness and grain size, we plotted the surface roughness Ra against the average grain size, as shown in Figure 4i, revealing a significant positive correlation (correlation coefficient R = 0.84). In conclusion, the surface roughness of films was directly proportional to the grain size, specifically increasing with larger grain size [37]. It is also demonstrated that Cu effectively inhibited the growth of grains and promoted the formation of smooth surfaces.

### 3.4. Optical Properties

**Figure 5:**
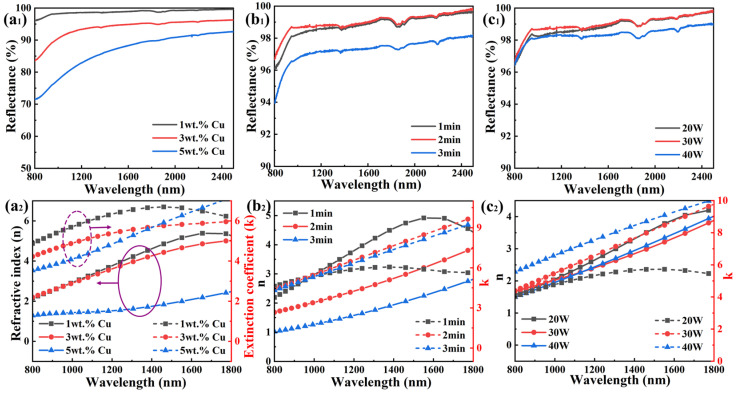
Optical properties of AgInCu_x_ thin films: (**a_1_**–**c_1_**) reflectance and (**a_2_**–**c_2_**) refractive index (n, solid lines) and extinction coefficient (k, dashed lines) of films varying Cu concentration, deposition time, and sputtering power, respectively.

Figure 4 shows the optical performance of AgInCu_x_ films deposited at various parameters, revealing their excellent reflectivity. The optical performances of AgInCu_x_ (x = 1, 3, and 5 wt.%) films are shown in Figure 5(a_1_,a_2_). The AgInCu exhibited the highest reflectivity of 99.66% during near-infrared wavelength. As the concentration of Cu increased, the refractive index and reflectance of films decreased. This decline was attributed to the increased optical loss resulting from a high dielectric constant. According to the Drude model, the dielectric constant consists of a real part ε_1_ and an imaginary part ε_2_ [25,29]:

ε = ε_1_ + iε_2_, (1)

ε_1_ = 1 − [ω_p_^2^/(ω^2^ + γ^2^)), (2)

ε_2_ =(ω_p_^2^γ)/(ω^3^ + γ^2^ω), (3)

where ω is the frequency of electromagnetic radiation, ω_p_ is the plasma frequency, and γ refers to the damping constant. The introduction of Cu atoms into the crystal lattice could induce local lattice distortions, impeding charge carrier transport and enhancing phonon scattering. As a result, the Hall mobility decreased. This increases the dielectric constant of films, typically manifesting as an increased imaginary component. The increased imaginary part signified the enhanced light absorption, ultimately leading to the reduction in reflected light intensity [29,38]. Therefore, lower Cu concentrations are favored for achieving optimal optical performance in AgInCu_x_ films.

Figure 5(b_1_,b_2_) presents the refractive index and extinction coefficient of AgInCu films deposited for different times. The AgInCu film deposited for 2 min displayed the highest reflectivity of 99.86% and average reflectance of 96.22%. It was revealed that as the deposition time increased, the refractive index decreased while the extinction coefficient exhibited a non-linear trend, initially increasing and then decreasing. As the deposition time increased from 1 to 2 min, the reflectance increased slightly due to the diminution of light transmission and scattering caused by the formation of continuous films. However, a further extension of deposition time to 3 min resulted in large grain size and increased surface roughness, which enhanced the light scattering [39]. Furthermore, the increasing extinction coefficient indicated that the absorption of light increased as the grain size increased, which resulted in reduced reflectance. Therefore, the films deposited for moderate deposition time achieved optimal reflectivity.

As shown in Figure 5(c_1_), the AgInCu films prepared at a sputtering power of 30 W exhibited superior optical performance with the highest reflectance of 99.86% and an average reflectance of 96.22% across the 800–2000 nm. As sputtering power was increased, the reflectance first displayed a small upward trend, followed by a gradual decrease at higher sputtering power. Additionally, from Figure 5(c_2_), the refractive index showed an opposite trend with the increasing sputtering power. The reflectivity of film R_M_ can be calculated by [8]:

R_M_ = (n_0_n_S_ − N_F_^2^)/(n_0_n_S_ + N_F_^2^), (4)

where n_0_ refers to the refractive index of the air, n_S_ refers to the refractive index of the substrate, and N_F_ refers to the refractive index of reflective films. This indicates that reflectance is related to the refractive index. Under specific conditions, such as normal incidence and negligible absorption, an increase in refractive index can lead to a decrease in reflectance of films. The initial improvement in reflectivity was influenced by the large grain size and a concomitant reduction in the density of grain boundaries [15]. The light scattering is predominantly localized at the grain boundary. Large grain size resulted in few grain boundaries, reducing light scattering and enhancing reflection. However, with the further increase in power, a decline in reflectivity, and an increase in the extinction coefficient were observed, which were attributed to enhanced scattering light. As a previous demonstration, increasing power promoted grain growth, which in turn elevated surface roughness. According to Mie scattering theory, the high roughness and large grain size amplified light scattering, leading to the increased extinction coefficient and decreased reflectivity [40]. As a result, moderate sputtering power was beneficial to improve the reflectivity of films.

### 3.5. Thermal Stability

**Figure 6:**
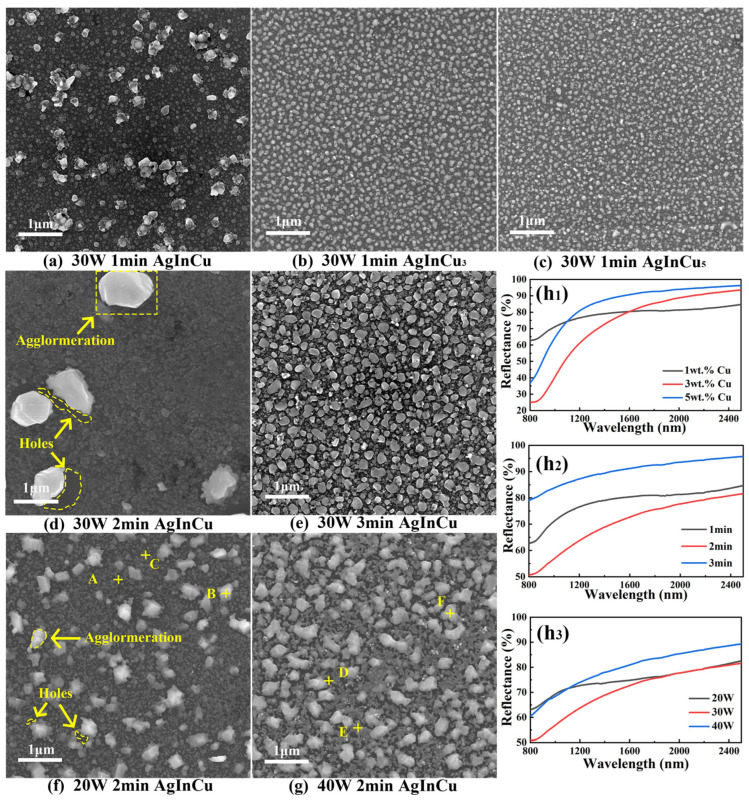
The (**a**–**g**) surface morphology and (**h_1_–h_3_**) reflectance of AgInCu_x_ films after exposure at 200 °C for 1 h.

**Figure 7:**
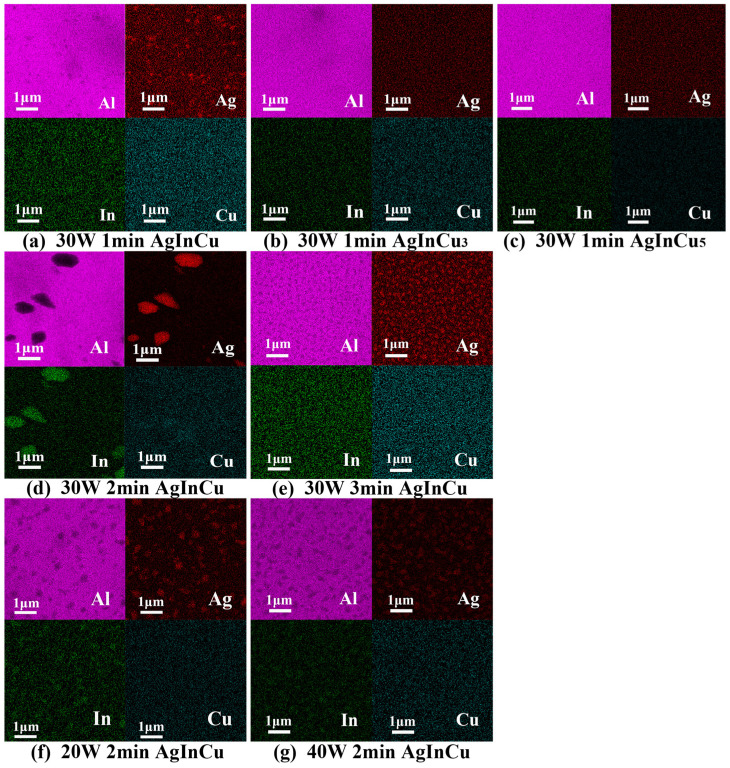
The (**a**–**g**) element mapping of AgInCu_x_ films after exposure at 200 °C for 1 h.

The surface morphology and reflectance of films after exposure at 200 °C for 1 h are shown in Figure 6. Corresponding elemental mapping by EDS is shown in Figure 7 with quantitative point analysis listed in Table 3. According to the SEM images of Figure 6a–g, particles and holes with varying sizes were observed on the film surfaces, disrupting their uniformity and continuity. EDS analysis of Table 3 and Figure 7a–g confirmed the particles and holes were inhomogeneous. There was an obvious difference in the chemical composition distribution between the particles and holes due to the agglomeration of grains. During the agglomeration, reduced activation energy for atomic migration at grain boundaries promotes atomic diffusion along the grain boundaries, leading to the formation of larger particles. Moreover, vacancy nucleation occurs at the film-substrate interface, followed by vacancy diffusion and aggregation, resulting in the formation of holes. [41,42]. Comparing the films with different Cu content from Figure 6a–c, as the copper content increased, the agglomerated particles exhibited a more uniform size distribution, and the average grain size decreased. This observation indicated that the addition of Cu served to inhibit the agglomeration of atoms. Roh et al. [43] showed that alloying the Ag layer with suitable elements enhanced thermal stability by reducing the surface free energy and suppressing Ag diffusion, thereby hindering agglomeration. Therefore, copper was an effective material for enhancing the thermal stability of films by inhibiting the agglomeration of atoms. Moreover, at long deposition time (Figure 6e) and high sputtering power (Figure 6g), the agglomerated particles exhibited a uniform size distribution. In contrast, the particles and holes were irregular at short deposition time (Figure 6a) and low sputtering power (Figure 6f). It was due to the high specific surface area and surface energy of small grains, which enhanced the diffusion and absorption of atoms, leading to the growth of irregularly shaped particles and holes.

The reflectance of films after exposure at 200 °C for 1 h is illustrated in Figure 6(h_1_–h_3_). A significant reduction in reflectance was observed in the AgInCu_x_ films after heat exposure. This reduction was attributed to the formation of discontinuous films resulting from surface agglomeration. Notably, an increase in Cu content correlated with a diminished decline in the reflectance of films after heat exposure. This trend was consistent with the findings from SEM results, further substantiating the hypothesis that the addition of Cu was beneficial to enhance the thermal stability of films through inhibiting the atomic agglomeration and improving the uniformity of films. Moreover, the decreases in reflectivity of the films were minimized at high sputtering power (P = 40 W) and deposition time (t = 3 min). This was attributed to the increased surface uniformity and reduced surface roughness, which effectively minimized light scattering and enhanced the reflectivity of films.

## 4. Conclusions

In summary, AgInCu_x_ (x = 1, 3, and 5 wt.%) films were deposited on an Al 6061 alloy substrate at different parameters using magnetron sputtering. Increasing Cu content reduced surface free energy and inhibited the migration of atoms to improve the thermal stability of films, although it led to the decline of reflectivity for the increasing light absorption. Therefore, the AgInCu_5_ films exhibited excellent thermal stability. Additionally, with increasing deposition time and sputtering power, the reflectance of the films initially increased and then decreased. The initial increase can be attributed to the improved continuity of the films, while the subsequent decrease is due to enhanced light scattering caused by larger grain sizes and increased surface roughness. Therefore, the AgInCu film deposited at a sputtering power of 30 W and a deposition time of 2 min exhibited the highest reflectance, achieving a peak value of 99.66% and an average reflectance of 96.22% across the near-infrared wavelength range.

## Figures and Tables

**Table 1:** The chemical composition of AgInCu_x_ targets (wt.%).

Targets	Ag	In	Cu
AgInCu	98	1	1
AgInCu_3_	96	1	3
AgInCu_5_	94	1	5

**Table 2:** The parameters of Ag alloy film deposited on the Al substrate.

Sample	Sputtering Power (W)	Power Density (W/cm^2^)	Deposition Time (min)	The Content of Cu (wt.%)	Thickness (nm)
1#	30	1.53	1	1	32.0 ± 0.6
2#	30	1.53	1	3	65.8 ± 2.8
3#	30	1.53	1	5	62.9 ± 1.8
4#	30	1.53	2	1	51.6 ± 0.5
5#	30	1.53	3	1	103.5 ± 0.8
6#	20	1.02	2	1	44.0 ± 0.5
7#	40	2.04	2	1	69.5 ± 0.4

**Table 3:** Point composition analysis of the corresponding positions in Figure 2.

Point	Element (wt.%)			
	Al	Ag	In	Cu
A	93.8	5.8	0.0	0.4
B	77.8	21.8	0.1	0.4
C	95	4.7	0.0	0.4
D	97	2.6	0.0	0.4
E	90.5	9	0.1	0.3
F	83	16.7	0.0	0.4

## Data Availability

The original contributions presented in this study are included in the article. Further inquiries can be directed to the corresponding authors.
